# Life-cycle assessment of transportation biofuels from hydrothermal liquefaction of forest residues in British Columbia

**DOI:** 10.1186/s13068-018-1019-x

**Published:** 2018-02-03

**Authors:** Yuhao Nie, Xiaotao Bi

**Affiliations:** 1 Department of Chemical and Biological Engineering, University of British Columbia, 2360 East Mall, Vancouver, BC V6T 1Z3 Canada; 2 Clean Energy Research Center, University of British Columbia, 2360 East Mall, Vancouver, BC V6T 1Z3 Canada

**Keywords:** GHG emissions, Life-cycle assessment, Hydrothermal liquefaction, Transportation biofuels, Forest residues, British Columbia

## Abstract

**Background:**

Biofuels from hydrothermal liquefaction (HTL) of abundantly available forest residues in British Columbia (BC) can potentially make great contributions to reduce the greenhouse gas (GHG) emissions from the transportation sector. A life-cycle assessment was conducted to quantify the GHG emissions of a hypothetic 100 million liters per year HTL biofuel system in the Coast Region of BC. Three scenarios were defined and investigated, namely, supply of bulky forest residues for conversion in a central integrated refinery (Fr-CIR), HTL of forest residues to bio-oil in distributed biorefineries and subsequent upgrading in a central oil refinery (Bo-DBR), and densification of forest residues in distributed pellet plants and conversion in a central integrated refinery (Wp-CIR).

**Results:**

The life-cycle GHG emissions of HTL biofuels is 20.5, 17.0, and 19.5 g CO_2_-eq/MJ for Fr-CIR, Bo-DBR, and Wp-CIR scenarios, respectively, corresponding to 78–82% reduction compared with petroleum fuels. The conversion stage dominates the total GHG emissions, making up more than 50%. The process emitting most GHGs over the life cycle of HTL biofuels is HTL buffer production. Transportation emission, accounting for 25% of Fr-CIR, can be lowered by 83% if forest residues are converted to bio-oil before transportation. When the credit from biochar applied for soil amendment is considered, a further reduction of 6.8 g CO_2_-eq/MJ can be achieved.

**Conclusions:**

Converting forest residues to bio-oil and wood pellets before transportation can significantly lower the transportation emission and contribute to a considerable reduction of the life-cycle GHG emissions. Process performance parameters (e.g., HTL energy requirement and biofuel yield) and the location specific parameter (e.g., electricity mix) have significant influence on the GHG emissions of HTL biofuels. Besides, the recycling of the HTL buffer needs to be investigated to further improve the environmental performance of HTL biofuels.

**Electronic supplementary material:**

The online version of this article (10.1186/s13068-018-1019-x) contains supplementary material, which is available to authorized users.

## Background

In British Columbia (BC), transportation consumes nearly 85% of total refined petroleum fuels [1] and generated about 25 million tonnes of carbon dioxide equivalent (CO_2_-eq) in 2014, which corresponds to approximately 38% of total greenhouse gas (GHG) emissions and leads all other economic sectors [2]. To address the concerns of global warming, BC government released its Climate Action Plan in 2008 and set up stepwise GHG emission reduction targets. The interim and ultimate targets aim at achieving 33 and 80% GHG reduction below 2007 levels by 2020 and 2050, respectively [3]. Besides the improvements in technology and operation efficiencies of transportation, displacing fossil fuels with biofuels is expected to make important contributions to reducing the GHG emissions.

Forest residues from logging operations, which contain branches, barks, tree tops, etc., are generally of no merchantable value and are burned as part of the forestry management strategy in BC [4]. The total volume of woody biomass available for bioenergy production in BC in 2016 was estimated to be around 21 million m^3^, of which 15.7% is forest logging residues [5]. Forest residues make up of 5–10% of the feedstock of BC wood pellet industry, which produces about 2 million tonnes of pellets annually, representing 61% of the total capacity of Canada [6]. However, 84% of the wood pellets produced end up being exported to Europe for district/home heating and power generation because of a lack of local markets [7, 8]. In BC, residential heating is mostly done by electricity and natural gas (NG), and more than 90% power is generated from hydro [9]. Besides, according to Pa et al. [10], the long-distance transportation of pellets could also result in a high-carbon footprint (295 kg CO_2_-eq/tonne of pellets). Therefore, one of the potential applications of abundant forest residues in BC is to produce liquid transportation fuels, such as gasoline, jet fuel, diesel, and heavy oil.

Hydrothermal liquefaction (HTL) of forest residues to intermediate product bio-oil with subsequent upgrading to finished products is one of the promising conversion pathways for transportation biofuel production. HTL decomposes biomass in subcritical to nearly critical water under moderate temperature (280–370 °C) and high pressure (10–25 MPa) [11], thus avoiding the pre-drying step in the conventional gasification and pyrolysis [12, 13]. Moreover, in contrast to pyrolysis, HTL can produce high quality and stable bio-oil with lower oxygen content (5–15 wt%) [14] and higher heating value (30–37 MJ/kg) [11], which has the potential to be directly co-processed with crude oil in a refinery [15–17]. In 2016, Licella reported that their HTL facility has successfully demonstrated the conversion of wood and agriculture wastes at commercial scale [18], and recently, it announced to collaborate with Canfor, a Canadian forest product company, to form a joint venture to integrate its HTL technology with Canfor’s pulp mills in Prince George, BC, to convert woody biomass to biofuels [19].

Many current researches on biofuels from HTL are trying to solve the technical obstacles, e.g., the design of HTL reactor for scale up [20], the integration of HTL with other systems [21], process parameter optimization [22] and the co-upgrading potential of HTL bio-oil with crude oil [16, 23], etc. There has been little investigation of HTL [17, 24] in terms of its GHG emission performance in comparison with gasification [25–30] and pyrolysis [17, 24, 30–37], based on a systematic review of the state-of-art life-cycle assessment (LCA) studies performed to quantify the GHG emissions of transportation biofuels from lignocellulosic biomass. The criteria of selecting the literatures for review are as follows: (1) only studies assessing thermochemical conversion technologies, i.e., pyrolysis, gasification, and HTL were included; (2) only studies focused on the lignocellulosic biomass were included, because this type of feedstock is abundantly available in BC with an established supply chain. Other types of feedstock like oil seed or algae are either unsustainable over short term nor short of stable supply; and (3) only LCA studies on the following liquid transportation fuels were included: jet, gasoline, and diesel, while ethanol was not considered. The detailed information of the reviewed studies is summarized in Additional file 1. According to the review, the following observations can be derived: (1) HTL is a promising conversion pathway in terms of GHG emission performance and (2) the results of LCA varied from study to study, due to the variation in geographic locations, settings of system, feedstock, analytical methods, and the treatment of by- or co-products. A review study focused on the pyrolysis technologies by Roy and Dias [38] has reported similar observations on the variability of LCA results based on feedstock, technology, etc.

In view of the great interest in deploying HTL technology to convert abundant but under-utilized forest residues in BC to biofuels, a specific LCA is timely needed to quantify its environmental impact. To our best knowledge, there has been no similar LCA study of HTL biofuels from forest residues based on BC context. Therefore, the results from this study could help provide a preliminary insight for other researchers and local companies or investors as well as a reference for government policy makers.

The following points were addressed in this study: (1) quantification of the life-cycle GHG emissions of HTL biofuel system based on different scenarios; (2) identification of the “hot spots” of the life-cycle processes that intensively emit GHGs; (3) analysis of the large proportional change of GHG emissions under different scenarios; (4) comparison of the GHG emissions of HTL biofuels produced in BC with general values reported in the literatures; (5) sensitivity analysis on the key parameters impacting the GHG emissions of HTL biofuels; and (6) making recommendations for improving the GHG emission performance of HTL biofuels.

## Methods

### Description of case study and processes

**Fig. 1:**
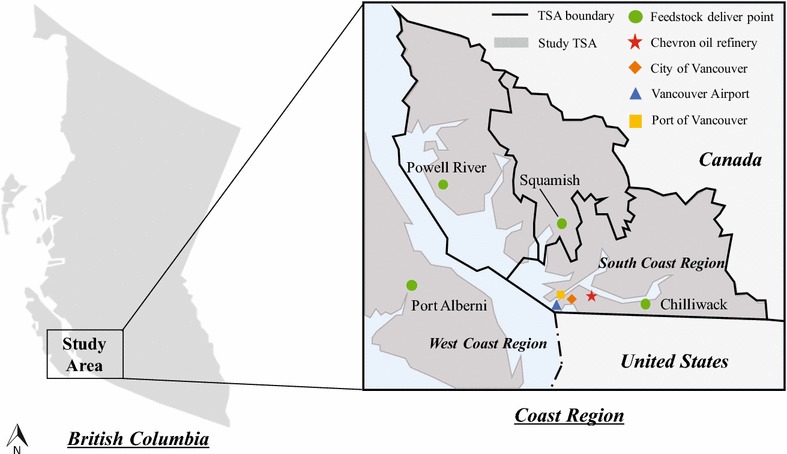
Schematic diagram of geographic information of HTL biofuels system (Powell River, Squamish and Chilliwack lie in the South Coast Region; Port Alberni lies in the West Coast Region)

A total liquid biofuel production rate of 100 million liters per year (MLPY) was assumed as the basis for this study, as proposed in an UBC–Boeing joint project report on evaluating the viability of bio-jet fuel production in western Canada, based on the availability and distribution of forest residues in BC [4]. The Coast Region of BC (see Fig. 1) was chosen as the study area for deploying the 100 MLPY hypothetic HTL biofuels system due to the abundantly available forest residues as feedstock, existing oil refining infrastructure for bio-oil upgrading, and local markets for biofuel product consumption in this area. BC government partitions the Coast Region into several timber supply areas (TSAs) and a sales office is established for timber marketing in each TSA. The locations of these timber sales offices, namely, Chilliwack, Squamish, Powell River, and Port Alberni, where biomass feedstock was assumed to be supplied to the conversion facilities, are referred to as the feedstock delivery points (FDPs) in this study. There is an existing Chevron oil refinery in Burnaby with a throughput of 8700 m^3^/days [39], and we assumed that this oil refinery was utilized to upgrade the bio-oil produced in HTL biorefinery. Four different biofuel products: gasoline, jet fuel, diesel, and heavy oil are produced and distributed to local markets for end use, specifically, gasoline and diesel for light- and heavy-duty vehicles, respectively, at City of Vancouver, jet fuel for airplanes at Vancouver International Airport, and heavy oil for marine vessels at Port of Vancouver. The geographic locations of all the places mentioned above are schematically shown in Fig. 1.

The forest residues availability for each TSA was estimated using the method proposed by MacDonald et al. in a BC government report [40]. The essence is to multiply the annual log harvest volume in that TSA by a biomass ratio, which is defined as the volume of forest residues recovered from unit volume of merchantable logs harvested in the logging operation. The annual log harvest volume was retrieved from the Harvest Billing System of BC based on the 5-year average data of August 2011–July 2016 [41]. In this study, we assumed that the biomass ratio was 15% based on the situation that most of the timbers in the BC Coast Region are the second growth Hemlock and the harvesting mode is ground-based and cable [40]. In ground-based harvesting systems, a machine travelling over the ground is used to carry the fell trees or logs from the stump to the landing. While in cable systems, the fell trees or logs are carried by a stationary machine with an overhead cable attached [42]. The density (dry basis) and moisture contents (wet basis) of forest residues used in this study are 420 kg/m^3^ and 49 wt%, respectively. Due to the lack of specific feedstock analysis data, i.e., proximate analysis and ultimate analyses, for forest residues in the Coast Region of BC, the feedstock analysis data from Tews et al. [17] were used in our models. The 5-year average annual volumes of harvest logs and estimated available forest residues are shown in Table 1.

**Table 1:** Annual forest residues availability in BC Coast Region

	Harvest logs (m^3^/year)	Biomass ratio	Forest residues availability		
			m^3^/year	Dry tonne/year	Wet tonne/year
Chilliwack	1.21E+06	0.15	1.82E+05	7.64E+04	1.50E+05
Squamish	4.98E+05	0.15	7.47E+04	3.14E+04	6.14E+04
Powell River	1.93E+06	0.15	2.89E+05	1.21E+05	2.38E+05
Port Alberni	5.24E+06	0.15	7.85E+05	3.30E+05	6.46E+05
Total	8.87E+06	0.15	1.33E+06	5.59E+05	1.09E+06

**Fig. 2:**
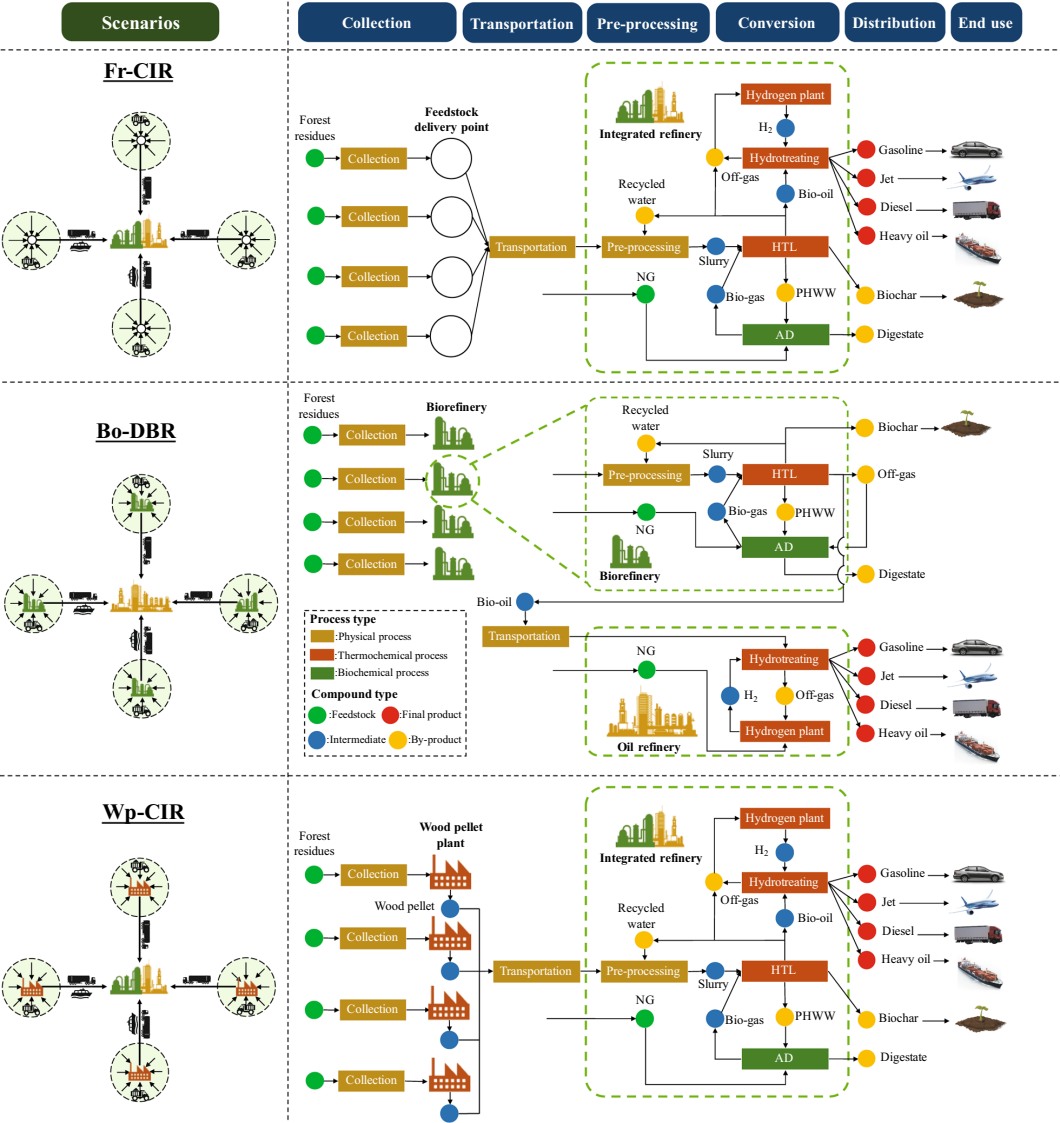
System configuration schematic and boundary of different HTL biofuel scenarios (AD, anaerobic digestion; NG, natural gas; PHWW, post HTL waste water)

The case study was developed based on three different scenarios. Although the processes vary with each scenario, the realm is from well to wheel. The basic structure of the HTL biofuel system includes the following stages: biomass feedstock collection and transportation, pre-processing, biomass-to-biofuel thermochemical conversion, and biofuel product distribution and end use. The main differences between these three scenarios lie in the configuration of biorefinery (integrated with oil refinery or distributed at FDPs) and the type of feedstock (bulky forest residues or forest residues derived bio-oil or wood pellets) supplied to refinery for conversion. For scenario 1 (denoted as Fr-CIR scenario), the collected bulky forest residues from each FDP are directly transported to the central integrated refinery for conversion. For scenario 2 (denoted as Bo-DBR scenario), forest residues are first converted to bio-oil at distributed biorefineries and then transported to a central oil refinery for upgrading. For scenario 3 (denoted as Wp-CIR scenario), forest residues are first densified to wood pellets at distributed pellet plants located at FDPs and then transported to the central integrated refinery for conversion. The system configuration schematic and boundary of each scenario are shown in Fig. 2.

#### Description of case study and processes: biomass collection

Biomass collection stage is the same for three scenarios and was modeled by two steps. The piled forest residues on forest stands of each TSA are first gathered, chipped to smaller size and loaded to dump trucks, and then shuttled to the FDP. Due to the lack of specific location and productivity of each forest stand in the corresponding TSA, we simply assumed that the forest residues after logging operation were uniformly distributed around the FDP, and 12.5 km was used as the average distance for shuttling.

To meet the 100 MLPY biofuel production target, for Fr-CIR and Bo-DBR scenarios, a total of ~ 300,000 dry tonnes of forest residues are needed. While for Wp-CIR scenario, due to the consumption of forest residues as drying fuel in pellet plants, additional ~ 36,000 dry tonnes are required. The detailed methods of calculating the annual forest residues supply for different scenarios are given in Additional file 2.

#### Description of case study and processes: transportation

The transportation of biomass feedstock from FDPs to conversion facility varies with scenarios. For Fr-CIR scenario, forest residues arriving at FDPs are reloaded to semi-trailers (STs) and then directly transported to the central integrated refinery for conversion. However, for Bo-DBR and Wp-CIR scenarios, the arriving forest residues are first converted to bio-oil and wood pellets in the satellite HTL and pellet plants at FDPs, respectively, and then, the intermediate products are loaded to STs or liquid tanker trucks (LTTs) and transported to the refinery for further conversion. It should be noted that transportation from Powell River and Port Alberni to Chevron oil refinery will undergo marine routes. STs or LTTs were thus assumed to be carried by ferries run by British Columbia Ferry Services Inc. To account for the emission associated with carrying STs or LTTs and the feedstock, the total emissions of ferry transportation were allocated by the mass of people, semi-trailers or LTTs, and other vehicles, which are estimated by the information provided on the website of British Columbia Ferry Services Inc. [43]. The transportation distance from Chilliwack, Squamish, Powell River, and Port Alberni to Chevron oil refinery are 102, 74, 179 (including 37 km marine transportation), and 170 km (including 57 km marine transportation), respectively.

To minimize the transportation emission, the total feedstock requirement is apportioned among four FDPs according to their feedstock availability and proximity to Chevron oil refinery, that is, the closer the FDP to the refinery, the higher priority it will be given for forest residues utilization. The detailed data for the annual forest residues transported from each FDP to the conversion facility can be found in Additional file 2.

#### Description of case study and processes: pre-processing

For biomass feedstock pre-processing in biorefinery, the incoming forest residues or wood pellets are first unloaded, cleaned, and sent to a grinder for further size reduction. Then, the ground feedstock is mixed with hot water recycled from HTL reaction, producing biomass–water slurry with 8 wt% solids content [17]. To make the life-cycle stages consistent between different scenarios, we also incorporated pellet plant operation in Wp-CIR scenario into the pre-processing stage. For pellet plant operation emission modeling, we used the data from Pa et al. [10], which can be found in Additional file 3.

#### Description of case study and processes: conversion

Conversion stage includes HTL of biomass feedstock, subsequent hydrotreating of bio-oil, and anaerobic digestion (AD) and hydrogen production processes, as depicted in Fig. 2. The AD unit was integrated with HTL system to treat and recover energy from the HTL wastewater, and a hydrogen production process was designed to meet the hydrogen demand for bio-oil hydrotreating. Both HTL and AD processes are located in the biorefinery, while hydrotreating and hydrogen production processes are located in the oil refinery. The parameters used for modeling the processes in biorefinery and oil refinery are given in Tables 2 and 3, respectively.

**Table 2:** Major inputs and parameters for modeling HTL biorefinery processes

Parameters	Value	References
Annual operating hours, h	8000	
Hydrothermal liquefaction		
Material and energy input		
Buffer (Na_2_CO_3_) content, wt% of slurry	1	[44]
Electricity, MW	4.03^a^/4.10^b^	Scaled from [17]
Heat, MW	50.42^a^/50.24^b^	Scaled from [20]
Products yields, kg/kg dry feedstock^c^		[17]
Bio-oil	0.367	
Off-gases	0.173	
Water (with dissolved organics)	0.404	
Biochar	0.056	
Off-gases composition, wt%		[17]
CO_2_	90.2	
H_2_	0.9	
CH_4_	3.0	
C_2_H_6_	2.5	
C_3_H_8_	1.9	
C_4_H_10_	1.5	
Anaerobic digestion		
Products yield, kg/kg wastewater		[17]
Biogas	0.23	
Solid digestate	0.01	
Liquid digestate	0.76	
Material and energy input		Average of [46, 47]
Electricity, MJ/GJ biogas produced	102.32	
Heat, MJ/GJ biogas produced	140.89	

^a^This value is applicable to Fr-CIR and Bo-DBR scenarios. For Bo-DBR scenario, this is the total electricity/heat input of the HTL units of four distributed biorefineries

^b^This value is applicable to Wp-CIR scenario

^c^Feedstock stands for either forest residues or wood pellets, and wood pellets were assumed to have the same conversion rate as forest residues

**Table 3:** Major inputs and parameters for modeling oil refinery processes

Parameters	Value	References
Annual operating hours, h	8000	
Hydrotreating		
LHSV, h^−1^	0.22	[17]
Material and energy input		
H_2_, g H_2_/g dry bio-oil	0.033	[44]
Electricity, MW	1.12	Scaled from [17]
Catalyst		
Load, kg catalyst/tonne bio-oil	0.41	Calculated based on LHSV
Life, years	1	
Products distribution, wt%		[17]
Deoxygenated oil	75	
Water	18	
Off-gases	7	
Off-gases composition, wt%		[17]
H_2_	7.8	
CH_4_	18.2	
C_2_H_6_	15.1	
C_3_H_8_	13.2	
C_4_H_10_	4.9	
C_5_H_12_	1.5	
C_6_H_14_	39.3	
Deoxygenated oil distillation streams, wt%		[23]
Gasoline	21	
Jet	25	
Diesel	35	
Heavy oil	19	
Hydrogen plant		
GHSV, h^−1^	4000	[51]
Material and energy input		
NG (feedstock), kg/m^3^ H_2_ produced	0.24	Scaled from [50]
Steam (feedstock), kg/m^3^ H_2_ produced	0.76	Scaled from [50]
NG (fuel), kg/m^3^ H_2_ produced	0.03	Scaled from [50]
Catalyst		
Load, kg catalyst/tonne H_2_ produced	0.12	Calculated based on GHSV
Life, years	3	
Electricity, MW	0.15	Scaled from [50]

The HTL process in this study was modeled based on the experimental and simulation data from PNNL report by Tews et al. [17]. The water–biomass slurry generated in the pre-processing process is pressurized and sent to the HTL reactor. The HTL process produces bio-oil, non-condensable gases, biochar as well as water containing high concentration of dissolved organics, called post HTL waste water (PHWW). Sodium carbonate (Na_2_CO_3_) is used as the buffer agent to prevent the pH of the slurry from dropping below 4, thus inhibiting the formation of high molecular weight compounds and solid wastes [44]. HTL bio-oil has low oxygen content and is thermally stable [17]. Therefore, we assumed that it was directly co-processed with crude oil in the Chevron oil refinery without pre-hydrotreating step. Non-condensable gases, referred to as off-gases in this study, contains the non-condensable volatile compounds, mostly CO_2_, a moderate part of light hydrocarbons (C_1_–C_4_), and a small portion of H_2_ (see Table 2). The energy in off-gases was assumed to be recovered and reused in conversion processes. For Fr-CIR and Wp-CIR scenarios, off-gases are sent to hydrogen plant as fuel for hydrogen production, and the remaining is used as fuel for heating anaerobic digester. For Bo-DBR scenario, these gases cannot be sent to hydrogen plant, so they are consumed as heating fuel for HTL and AD. It was assumed that the carbon released from the utilization of these off-gases did not contribute to climate change, since it essentially origins from the carbon intake during tree growth. The solid-phase product biochar was assumed to be collected and applied for soil amendment in local farms, which will be described in the HTL biofuel LCA section below. Panisko et al. [45] reported that chemical oxygen demand (COD) of PHWW ranged from 41,000 to 77,000 mg/L, compared with 200 to 1200 mg/L of raw municipal wastewater. Hence, a dedicated treatment facility must be employed at the processing facility. In this study, we assumed that an AD unit was designed for the treatment and energy recovery of PHWW, while the majority of PHWW was recycled for slurry formation in the biomass feedstock pre-processing.

In the anaerobic digester, the PHWW is converted into biogas and solid and liquid digestates. Biogas is sent to the HTL unit as heating fuel. The solid and liquid digestates are sent to landfill and wastewater treatment plant, respectively, for further treatment, but the impact of both processes is outside the scope of this study. Due to the lack of reported data for PHWW as substrate for AD, a large-scale AD operating at mesophilic temperature (35 °C) and using liquid swine manure as feedstock was used as an approximation to quantify the heat and electricity requirements [46, 47]. A typical large-scale AD can digest 20,000–60,000 tonnes of raw materials per year [47]. The PHWW input into the AD unit in this study is ~ 409,000 tonnes per year (see Additional file 2), which is about ten times larger. While the world’s largest AD plant reported in 2013 being constructed digests 270,000 tonnes organic wastes per year [48], no energy input data of this plant are available. The energy input of a typical large-scale AD is thus the best available data to be used in our study.

The hydrotreating process is a catalytic reaction process, where the oxygenated compounds in bio-oil are exposed to hydrogen under elevated pressure and high temperature [49]. The catalyst utilized in hydrotreating process was assumed to be conventional NiMo/Al_2_O_3_ catalyst which is commonly used in crude oil hydroprocessing. The effluent from hydrotreating reactors is cooled and separated into deoxygenated oil, wastewater, and off-gas streams. The off-gases from hydrotreating containing mainly light hydrocarbons (see Table 2) are sent to hydrogen plant as feedstock for steam reforming. The deoxygenated oil is then distilled into gasoline, jet, diesel, and heavy oil as finished products.

Hydrogen for bio-oil upgrading is produced by NG steam reforming in a hydrogen plant of the oil refinery. The hydrogen demand was determined by the bio-oil production from HTL. As reported in the study of Zhu et al. [44], per gram of dry bio-oil to be treated, 0.033 g of H_2_ is needed. The hydrogen production process was modeled based on an NREL report by Spath et al. [50] with scaling to the specific hydrogen demand. Certain modifications were made on the NREL model to accommodate the entire HTL biofuel production system. Specifically, the reformer is fueled mainly by the combustible off-gases from hydrogen production, while the remaining 4.4 wt% [50] was assumed to be supplied by off-gases from hydrotreating as well as HTL depending on the scenarios, instead of using purchased NG. The electricity requirement of the hydrogen plant was modified to be met by BC grid. The catalyst utilized in hydrogen production process is assumed to be NiMo/Al_2_O_3_.

#### Description of case study and processes: distribution and end use of biofuels

Four different liquid biofuel products: gasoline, jet fuel, diesel, and heavy oil are produced in Chevron oil refinery and distributed to the local markets. The gasoline and diesel were assumed to be delivered by LTT to a hypothetic refueling station for light- and heavy-duty vehicles, respectively, at City of Vancouver, which is 10 km away from Chevron refinery. The jet fuel is delivered via an existing 40 km oil pipeline from Chevron oil refinery to Vancouver International Airport, for airplanes. The heavy oil is delivered by LTT to Port of Vancouver, 11.3 km away from Chevron refinery, for marine vessels.

### HTL biofuel LCA

An attributional LCA was conducted to quantify the GHG emissions of HTL biofuels from forest residues in BC. The analysis follows the international standard for LCA, ISO 14040 [52], and the functional unit is set to be 1 megajoule (MJ) of HTL biofuel mix produced.

All emissions from each process and its associated upstream supply chain were accounted for in this study. However, the emissions associated with construction of infrastructure, manufacture of equipment as well as waste water treatment were not included within the scope. In addition, forest residues as feedstock for biofuels production were considered to carry no environmental burdens linked with the harvested timber in light of low value of these forest residues, which otherwise will be burned to reduce the risk of wild fire in BC. We also assumed no soil carbon change due to controlled sustainable removal of forest residues from forest stands to produce biofuels in BC, with ~ 25% of forest residues being left in forest stands to provide nutrients and for the health of the forests [4].

The method of handling process by-products can significantly influence the life-cycle results of biofuel [30, 35, 53]. The by-product biochar produced in HTL plant was assumed to be shipped out to a hypothetic farm 50-km away from biorefinery and applied for soil amendment. HTL biochar contains carbon originating from forest residues and was modeled as sequestered carbon in this analysis. Although the stability of carbon in biochar depends on many factors such as feedstock, processing, and environmental conditions, we assumed that 80% of the carbon in biochar could be stably sequestrated when it is applied for soil amendment as suggested by Roberts et al. [54]. Wang et al. [55] meta-analyzed 24 studies of biochar decomposition in soil and found that about 97% of biochar could contribute to long-term carbon sequestration in soil. Hence, the 80% assumption we made in this analysis is conservative. Besides the sequestered carbon credit, N in biochar was assumed to displace the same amount of nitrogen fertilizer as suggested by Han et al. [35]. The emissions associated with the nitrogen fertilizer production are avoided, thus creating another credit. The average data were used to calculate the credit for replacing nitrogen fertilizer. The C and N contents in biochar were assumed to be 51 and 0.5 wt%, respectively [56].

Data quality and specificity are generally considered as critical issues for LCA studies. Spatial variation and local environmental uniqueness are one of the concerns that require special attention [57]. Therefore, to enhance the consistency and accuracy of the life-cycle inventory data, whenever possible, BC specific data were utilized, e.g., the feedstock availability, the locations of biofuel supply chain nodes, the transportation, and electricity mix. Otherwise, Canadian average, or if not available, US average, data were used with modification on the electricity mix to reflect BC specific mix. GHGenius v4.03 [58], a Canadian-based LCA program, was primarily employed for modeling processes such as transportation, energy and fuel production, and consumption, by setting BC as the analyzed region and 2016 as the base year. For the processes lacking built-in models in GHGenius v4.03, e.g., material production and delivery including HTL buffer agent, hydrotreating and hydrogen production catalyst, and nitrogen fertilizer, the GREET 2015 (Greenhouse gases, regulated emissions, and energy use in transportation) [59] or SimaPro v8.2 coupled with Ecoinvent v3.2 database [60] was used to model the process emissions with modification on the electricity mix. When the data could not be found in the software database described above, they are collected from peer-reviewed journal articles or reports issued by government and widely recognized scientific organizations (e.g., IPCC) or laboratories (e.g., PNNL and NREL).

The emissions from each process are obtained based on the emission factor method. Concretely, materials and energy consumptions were first calculated for each process through mass and energy balances and then multiplied by the corresponding emission factors. The detailed mass and energy balances of pre-processing and conversion stages can be found in Additional file 2. The equipment energy input and processes emission factors used in modeling are summarized in Additional file 3. The collected raw data from the various sources described above were first compiled in Microsoft Excel to build the life-cycle inventory of HTL biofuels, and then, IPCC 2007 global warming potential factors were used to convert CO_2_, CH_4,_ and N_2_O into CO_2_-eq for a time horizon of 100 years.

## Results and discussion

### Life-cycle GHG emissions

**Fig. 3:**
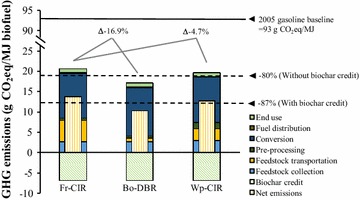
Stagewise GHG emissions of HTL biofuels from three different scenarios

Figure 3 shows the life-cycle stagewise GHG emissions of three different HTL biofuel production scenarios. The life-cycle GHG emissions for Fr-CIR, Bo-DBR, and Wp-CIR scenarios are 20.5-, 17.0-, and 19.5-g CO_2_-eq/MJ, respectively, corresponding to 78, 82, and 79% reduction relative to 2005 gasoline baseline 93-g CO_2_-eq/MJ [61]. When considering the credit from biochar applied for soil amendment, the life-cycle GHG emissions of HTL biofuels can be further reduced by 6.8-g CO_2_-eq/MJ, corresponding to 85, 89, and 86% reduction of the life-cycle GHG emissions compared to petroleum fuels for Fr-CIR, Bo-DBR, and Wp-CIR scenarios, respectively. The detailed GHG emission profile of individual processes is given in Table 4. For all three scenarios, the most dominant contributor to GHG emissions is biofuel conversion, which makes up more than 50%, followed by feedstock delivery, including collection and transportation of biomass feedstock, accounting for 19–39% of total emissions depending on specific scenario. The process having the highest global warming impact over the whole life cycle of HTL biofuels is the HTL buffer agent Na_2_CO_3_ production. In this study, due to the lack of industrial data of Na_2_CO_3_ consumption in the HTL process, we used the bench test data reported by Zhu et al. [44] and Panisko et al. [45] from PNNL, i.e., Na_2_CO_3_ was consumed at 1 wt% of the total feed slurry. This value could be higher than the demonstration- or industrial-scale data because of the assumed no-recycling of Na_2_CO_3_. Although we assumed that PHWW was recycled for the slurry formation, we did not consider the remaining Na_2_CO_3_ in the recycled waste water, because no data are currently available about the buffer consumption rate in the HTL reaction. The contribution of biomass collection is similar among the three scenarios (13–16%), while the transportation varied significantly. In Fr-CIR scenario, feedstock transportation accounts for about 25% to the GHG emissions of HTL biofuel. The long-distance transportation and the low energy density of bulky forest residues lead to the high transportation emissions. In contrast, for Bo-DBR and Wp-CIR scenarios, the bulky forest residues are first densified into high energy density intermediate products, bio-oil, and wood pellets, which are transported to refinery for further conversion. Compared with Fr-CIR scenario, Bo-DBR and Wp-CIR scenarios can lower the transportation emissions by 83 and 44%, respectively. However, the configuration change also causes increase of GHG emissions in other stages. For Wp-CIR scenario, due to the use of biomass feedstock as heating fuel in pellet plant operation, more forest residues need to be collected from forest stands, thus increasing the emissions of collection stage. Besides, pellet plant operation is linked with upstream (heating fuel production and electricity generation) and downstream (fuel combustion) emissions [10], which contribute to the increased emissions in pre-processing stage compared with the other two scenarios. For Bo-DBR scenario, the off-gases produced in the distributed HTL plants could not be used in the refinery as in integrated systems, i.e., Fr-CIR and Wp-CIR, so NG is needed as a feedstock for hydrogen production, thus increasing hydrogen production emissions. Overall, Bo-DBR and Wp-CIR scenarios can achieve 16.9 and 4.7% reduction of total GHG emissions compared with Fr-CIR scenario. In Fr-CIR and Wp-CIR scenarios, AD operation is an important contributor to the life-cycle GHG emissions of HTL biofuels, accounting for around 14%. NG is used as heating fuel for maintaining the AD operating temperature, since off-gases produced by HTL are sent to refinery for hydrogen production. However, in Bo-DBR scenario, the impact of AD operation can be considerably reduced due to the use of remaining off-gases from HTL as heating fuel for AD.

**Table 4:** Percent contribution of each process stage to the life-cycle GHG emissions of HTL biofuels

HTL biofuel life-cycle stage	Fr-CIR (%)	Bo-DBR (%)	Wp-CIR (%)
Feedstock collection	13.12	15.79	15.47
Loader and chipper operation	7.53	12.28	12.22
Forest residues shuttling to FDPs	5.59	3.51	3.25
Feedstock transportation	25.47	5.29	14.90
Pre-processing	2.88	3.47	8.07
Grinder and dust collector operation	2.25	2.71	2.36
Loader operation	0.63	0.76	0.66
Pellet plant operation	N/A	N/A	5.04
Conversion	53.36	69.23	56.11
Electricity	4.17	5.02	4.44
HTL buffer	34.44	41.46	36.15
AD gas combustion in HTL burner	1.10	1.32	1.15
AD operation	13.53	5.02	14.24
Hydrogen production	0.07	16.34	0.07
Hydrotreating catalyst	0.06	0.07	0.06
Fuel distribution	0.17	0.20	0.17
End use	5.00	6.02	5.28

### Comparison with peer-reviewed literatures

**Fig. 4:**
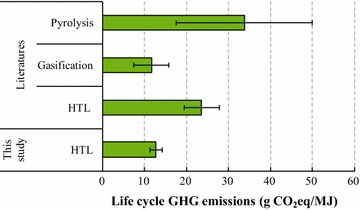
Comparison of HTL biofuel life-cycle GHG emissions results with literatures

To check whether the HTL biofuel GHG emission results from this study are consistent with those from peer-reviewed literatures, we have conducted a comparison using the results from part of the reviewed studies, as presented in Additional file 1. The studies were chosen for comparison only when the following criteria are met: (1) the system boundary is well-to-wheel or well-to-wake and (2) the functional unit is MJ of biofuel. Figure 4 presents the analyzed life-cycle GHG emissions of this study and those of literatures. The bar in Fig. 4 stands for the median value of the GHG emission results of all studies of a specific conversion pathway, instead of the mean value, because we found that the mean can be easily influenced by the extreme values of certain studies, and the error bar represents the standard deviation. It should be noted that the GHG emission results of this study used to compare are the net emission results including the biochar credit, since the results from majority of the selected literatures consider the by- or co-products credit.

According to the results shown in Fig. 4, gasification generally has the best GHG emission performance (11.6 ± 4.14 g CO_2_-eq/MJ), followed by HTL (12.67 ± 1.46 g CO_2_-eq/MJ from this study and 23.58 ± 4.18 g CO_2_-eq/MJ from literatures) and finally pyrolysis (33.77 ± 16.24 g CO_2_-eq/MJ). Our results seem to be consistent with the general trend, although it is about 46% lower than the median value of HTL biofuel results from the literatures. This could be explained by the much lower emission intensity of the BC electricity mix compared with the US electricity mix, which was used in the two HTL LCA studies we reviewed [17, 24]. Another major reason could be the credit assigned for by-products. In this study, we assumed that by-product biochar applied for soil amendment could create credits both from carbon sequestration in the soil as well as the avoidance of nitrogen fertilizer production. In contrast, the other two studies did not consider the credit from biochar produced by HTL.

### Sensitivity analysis

To investigate key factors influencing the GHG emissions of HTL biofuels, a sensitivity analysis was conducted by adjusting the nominal values of uncertain parameters to − 10 and + 10%. For electricity mix sensitivity analysis, BC electricity mix was entirely displaced with Alberta (AB) electricity mix with keeping all other modeling parameters unchanged. The parameters as well as their values used for sensitivity analysis are categorized and are listed in Table 5. It should be noted that the life-cycle GHG emissions are the net values with the biochar credit considered.

**Table 5:** List of parameters used for sensitivity analysis of GHG emissions of HTL biofuels

Category	Parameters	Nominal	− 10%	+ 10%
Feedstock property	Moisture content of forest residues: wt%	48.91	44.02	53.80
Feedstock supply	Feedstock collection distance: km	12.5	11.25	13.75
Process performance	Biomass content in slurry for HTL: wt%	8.0	7.2	8.8
	Bio-oil yield: kg/kg dry wood	0.367	0.330	0.404
	HTL energy requirement: MW	50.4	45.38	55.46
	Biofuel yield: kg/kg bio-oil	0.75	0.68	0.83
By-product credit	Carbon sequestered in biochar: wt%	80	72	88
Location specificity	Electricity mix: %			
	BC electricity mix^a^: hydro: 90.4; biomass: 4.9; NG: 2.9; fuel oil: 1.5; wind: 0.3			
	AB electricity mix^b^: Coal: 72.4; NG: 19.6; wind: 3.6; hydro: 3.5; fuel oil: 0.9			

^a^From [9], average of 2010–2012, detailed emission factors are shown in Additional file 3

^b^From [9], average of 2010–2012, detailed emission factors are shown in Additional file 3

**Fig. 5:**
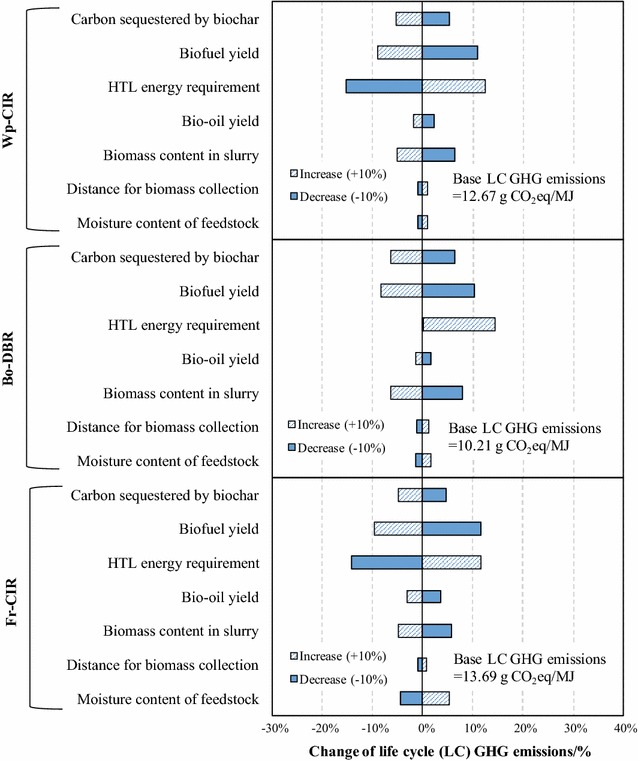
Sensitivity analysis of net life-cycle GHG emissions of HTL biofuels for different scenarios

As shown in Fig. 5, although each scenario presented different results, in general, the most sensitive parameters are associated with process performance. The change of HTL energy requirement and biofuel yield by 10% can lead to more than 10% variation of the GHG emissions for all scenarios. However, it should be noted that for Bo-DBR scenario, the 10% decrease of HTL energy requirement does not have appreciable impact on the GHG emissions. This is because at the nominal HTL energy requirement level, the HTL and AD units can be self-energized by biogas from AD as well as off-gases from HTL in Bo-DBR scenario. Therefore, further lowering the HTL energy requirement will not make a difference.

The 10% change in biomass content in slurry and carbon sequestrated in biochar shows a moderate impact (range of ± 5 to ± 10%). With a fixed biomass input, the biomass content in slurry can influence the total weight of slurry, which further determines the electricity consumption of pumping as well as the input of HTL buffer Na_2_CO_3_. As the results in Table 4 suggest, the consumption of Na_2_CO_3_ is a crucial contributor to the GHG emissions of HTL biofuels. Biochar, in this study, was assumed to be applied for soil amendment and created GHG credits from carbon sequestration as well as the avoidance of nitrogen fertilizer production. Although there is uncertainty regarding the biochar carbon stability in the soil, reported studies [55, 62] generally show a stable property of the biochar carbon. However, specific models need to be developed in the future to verify the carbon sequestration potential of HTL biochar.

The 10% change in other parameters such as bio-oil yield, moisture content of forest residues, and feedstock collection distance has modest (within ± 5%) impact on the GHG emissions. It should be mentioned that although moisture content of forest residues is considerably sensitive to Fr-CIR scenario, it makes little impact for Bo-DBR and Wp-CIR scenarios. Bio-oil yield does not influence the GHG emissions of HTL biofuels as much as biofuel yield, because the bio-oil yield has larger impact on the biochar credit, which can offset the impact of other life-cycle stages, than biofuel yield. The biochar credit is directly related to biochar yield, which can be influenced by bio-oil yield from two layers. First, the change of bio-oil yield can impact the feedstock requirement, which leads to parallel change of the yield of all HTL products, i.e., bio-oil, off-gases, biochar, and PHWW. The second layer is that the bio-oil yield can influence HTL products profile. For example, the decrease of bio-oil yield will increase the fraction of biochar in HTL products. In contrast, the change of biofuel yield only has the first layer effect.

**Fig. 6:**
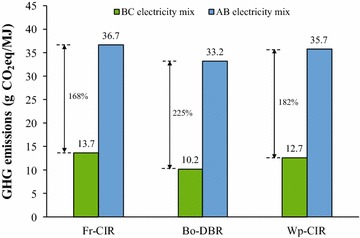
Sensitivity of electricity mix on net life-cycle GHG emissions of HTL biofuels for different scenarios

The significant impact of electricity mix on the HTL biofuel GHG emissions is indicated in Fig. 6. With more than 90% renewable composition, BC’s electricity mix is more favorable than that of AB. A change from BC electricity mix to AB electricity mix can lead to a 168, 225, and 182% increase in the GHG emissions for Fr-CIR, Bo-DBR, and Wp-CIR scenarios, respectively. Therefore, locating the potential HTL system in a place with clean electricity mix like BC can considerably lower the GHG emissions of HTL biofuels.

### Improving the GHG emission performance of HTL biofuels

Based on the life-cycle “hot spots” and the key parameters impacting the GHG emissions of HTL biofuels identified in this study, the following recommendations can be made to help improve the GHG emission performance of HTL biofuels produced in BC as well as to provide insights for companies or investors who want to deploy such a facility:

1. Increase the recycling rate of HTL buffer Na_2_CO_3_ with the understanding of energy and material consumption of the recycling process. Na_2_CO_3_ use has been identified to contribute mostly to the life-cycle GHG emissions of the proposed HTL plant in BC. According to our analysis, if the recycling rate of the buffer increases by 25%, the GHG emissions can be reduced by 13–17%. This is a promising way to further increase the environmental performance of HTL biofuels, while the energy and material input associated with the recycling need to be first clearly understood.
2. Lower the transportation emission by densifying biomass feedstock before transportation to conversion facilities. For long-distance transportation of feedstock with high moisture content, we recommend to first convert these raw materials into high energy density intermediate product such as bio-oil or wood pellets. If the infrastructure is available within a reasonable distance, it will be ideal to utilize such existing facility. Otherwise, the economics of constructing the new infrastructure, or alternatively purchasing the mobile conversion devices, needed to be first investigated.
3. Increase the process performance of the HTL biofuel system. Specifically, the main efforts need to be put on increasing the energy efficiency of HTL and maximizing the biofuel yield. This relies on the optimization of HTL system design, e.g., integrated with AD, to reduce the fossil energy input. Other improvement can be made in increasing the biomass content in the slurry. With the advancement of pump technology, transmission of large-scale biomass–water slurry would be feasible.
4. Make full use of the processe by-products, i.e., off-gases, biochar, and PHWW, to create GHG savings. Off-gases can be used as either heating fuel for different operation units or feedstock for hydrogen production to avoid the input of external NG. Biochar applied for soil amendment can create credits from both carbon sequestration and the avoidance of nitrogen production, while specific models need to be developed to verify the carbon sequestration potential of biochar to reduce the uncertainty. HTL can be integrated with an AD unit to recover energy from the PHWW, hence increase the energy efficiency of HTL.
5. Locate the HTL biofuel system in a place with favorable electricity mix. This can make a big difference, as shown in the comparison of the electricity mix of BC and AB.

## Conclusions

This study quantified the life-cycle GHG emissions of a hypothetic 100 MLPY HTL biofuel production system in British Columbia based on three different system configurations. The results suggest that compared with the conventional petroleum fuels, up to 89% GHG emission reduction can be achieved by HTL biofuels with the biochar credit considered. The conversion stage dominates the total emissions, contributing more than 50%. The process emitting most GHGs over the life cycle of HTL biofuels is HTL buffer production, resulted from the large amount of buffer consumed to maintain the pH of biomass slurry in the HTL process. Recycling of the HTL buffer thus needs to be further investigated to reduce the impact. Converting forest residues to bio-oil and wood pellets before transportation can significantly lower the transportation emission and contribute to the considerable reduction of the life-cycle GHG emissions of HTL biofuels. A sensitivity analysis indicates the importance of process performance parameters, such as HTL energy requirement and biofuel yield, as well as the location specific parameter such as the electricity mix. Therefore, the main efforts can be put on increasing the energy efficiency of HTL and maximizing the biofuel yield to further improve the GHG emission performance of HTL biofuels.

## Additional files

**Additional file 1.** Review of LCA studies.

**Additional file 2.** Mass and energy balances.

**Additional file 3.** Equipment energy input and process emission factors.

## Abbreviations

LCA: life-cycle assessment
GHG: greenhouse gas
HTL: hydrothermal liquefaction
BC: British Columbia
AB: Alberta
NG: natural gas
MLPY: million liters per year
TSA: timber supply area
FDP: feedstock delivery point
ST: semi-trailer
LTT: liquid tanker truck
AD: anaerobic digestion
PHWW: post HTL waste water
LHSV: liquid hourly space velocity
GHSV: gas hourly space velocity
