# Supplementary material for: Life-cycle assessment of transportation biofuels from hydrothermal liquefaction of forest residues in British Columbia
Source: Biotechnol Biofuels. 2018 Feb 3;11:23. doi: 10.1186/s13068-018-1019-x (PMC5797420; doi:10.1186/s13068-018-1019-x)
Supplement: Supplementary file 1 — Additional file 1. Review of LCA studies. [file 13068_2018_1019_MOESM1_ESM.pdf]

Additional file 1 - Review of LCA studies

Table 1. Review of LCA studies on thermochemical conversion of lignocellulosic biomass to transportation biofuels

Conversion pathway	Reference	Published year	Region	System boundary	Facility capacity	Functional unit (FU)	Software	Feedstock	Focused product	LC GHG Emissions (g CO_2eq/FU)
Gasification	[1]	2007	Europe	WTW	200 MW	MJ of fuel	E^3 database	FR	Diesel	4.8
	[2]	2008	US	WTWa	N/A	MJ of fuel	GREET	FR; CS	Jet	FR:11.6; CS:5.4
	[3]	2009	Europe	WTW	N/A	km	Excel	FR	Diesel	90
	[4]	2010	US	WTWa	300 barrel/day	MJ of fuel	GREET	FR; SG	Jet	FR:12.2; SG: 17.7
	[5]	2010	Europe	WTT	N/A	MJ of fuel	GREET	SG	Diesel	21.6
	[6]	2013	US	WTWa	3000 dry tonne /day	MJ of fuel	GREET	CS	Jet	10.1
Pyrolysis	[7]	2011	US	WTW	200 tonne/day	Hectare of land	GREET	CS	Gasoline	-2.99E+06
	[8]	2011	US	WTW	2000 dry tonne/day	MJ of fuel	GREET	FR	Gasoline	42.90
	[9]	2012	US	WTW	2000 dry tonne/day	MJ of fuel	SimaPro and GREET	FR	Gasoline and diesel	G: 39; D: 39
	[10]	2012	N/A	WTT	500 dry tonne /day	MJ of fuel	SimaPro	SRP	Gasoline and diesel	-50.54
	[6]	2013	US	WTWa	2000 dry tonne/day	MJ of fuel	GREET	CS	Jet	29.4[a]/22.1[b]
	[11]	2013	US	WTW	2000 dry tonne/day	MJ of fuel	GREET	FR; CS	Gasoline	FR: 38[c]; CS: (10[a]/-16[b])[d]
	[12]	2014	US	WTW	2000 dry tonne/day	MJ of fuel	SimaPro and GREET	LR&FT	Gasoline and diesel	G: 33.8; D: 34.0
	[13]	2014	N/A	WTT	2000 dry tonne/day	MJ of fuel	GREET	CS	Gasoline and diesel	28.82[e]; 25.15[f]; -18.13[g]
	[14]	2017	US	WTW	N/A	MJ of fuel	GREET	FR	Jet	22[h];37[i]
HTL	[12]	2014	US	WTW	2000 dry tonne/day	MJ of fuel	SimaPro and GREET	LR&FT	Gasoline and diesel	G: 27.2; D: 27.3
	[14]	2017	US	WTW	N/A	MJ of fuel	GREET	FR	Jet	18[h];20[i]

Note: WTWa=Well-to-wake; WTW=Well-to-wheel; WTT=Well-to-Tank; FR=Forest residue; CS=Corn stover; SG=Switchgrass; SRP=Short Rotation Polar; LR&FT=Logging residues and forest thinnings; G: gasoline; D: diesel;[a] Byproduct bio-char is used for power generation; [b] Byproduct bio-char is used for soil amendment; [c] H_2 is from fuel gas reforming; [d] H_2 is from pyrolysis oil reforming; [e] Hydrogen comes from external NG reforming, and biofuel yield is 43.5%; [f] Hydrogen comes from steam reforming of 35% bio-oil, and biofuel yield is 33.1%; [g] Hydrogen comes from steam reforming of 100% bio-oil, and biofuel yield is 16.1%; [h] In-situ hydrogen production via steam reforming of process off-gases; [i] Ex-situ hydrogen production via steam reforming of natural gas.

Reference

1. Edwards R, Larive J-F, Mahieu V, Rounveirolles P. Well-to-wheels analysis of alternative fuels and powertrains in the European context Version 2c. JRC. 2007.

2. Wong HM. Life-cycle assessment of greenhouse gas emissions from alternative jet fuels. Massachusetts Institute of Technology; 2008.

3. van Vliet OPR, Faaij APC, Turkenburg WC. Fischer-Tropsch diesel production in a well-to-wheel perspective: A carbon, energy flow and cost analysis. Energy Convers. Manag. Elsevier Ltd; 2009;50:855–76.

4. Stratton RW, Min Wong H, Hileman JI. Life cycle greenhouse gas emissions from alternative jet fuels. 2010;571:1–133.

5. Hoefnagels R, Smeets E, Faaij A. Greenhouse gas footprints of different biofuel production systems. Renew. Sustain. Energy Rev. 2010;14:1661–94.

6. Han J, Elgowainy A, Cai H, Wang MQ. Life-cycle analysis of bio-based aviation fuels. Bioresour. Technol. 2013;150:447–56.

7. Kauffman N, Hayes D, Brown R. A life cycle assessment of advanced biofuel production from a hectare of corn. Fuel. 2011;90:3306–14.

8. Han J, Elgowainy A, Palou-Rivera I, Dunn JB, Wang MQ. Well-to-wheels analysis of fast pyrolysis pathways with GREET. 2011.

9. Hsu DD. Life cycle assessment of gasoline and diesel produced via fast pyrolysis and hydroprocessing. Biomass and Bioenergy. 2012;45:41–7.

10. Iribarren D, Peters JF, Dufour J. Life cycle assessment of transportation fuels from biomass pyrolysis. Fuel. 2012;97:812–21.

11. Han J, Elgowainy A, Dunn JB, Wang MQ. Life cycle analysis of fuel production from fast pyrolysis of biomass. Bioresour. Technol. 2013;133:421–8.

12. Tews IJ, Zhu Y, Drennan CV, Elliott D., Snowden-Swan LJ, Onarheim K, et al. Biomass direct liquefaction options: technoeconomic and life cycle assessment. PNNL. Richland; 2014.

13. Dang Q, Yu C, Luo Z. Environmental life cycle assessment of bio-fuel production via fast pyrolysis of corn stover and hydroprocessing. Fuel. 2014;131:36–42.

14. de Jong S, Antonissen K, Hoefnagels R, Lonza L, Wang M, Faaij A, et al. Life-cycle analysis of greenhouse gas emissions from renewable jet fuel production. Biotechnol. Biofuels. 2017;10:64.
