# Supplementary material for: Life-cycle assessment of transportation biofuels from hydrothermal liquefaction of forest residues in British Columbia
Source: Biotechnol Biofuels. 2018 Feb 3;11:23. doi: 10.1186/s13068-018-1019-x (PMC5797420; doi:10.1186/s13068-018-1019-x)
Supplement: Supplementary file 2 — Additional file 2. Mass and energy balances. [file 13068_2018_1019_MOESM2_ESM.pdf]

Additional file 2 - Mass and energy balances

This file contains the following contents:

1. Calculation of annual forest residues requirement
2. Stream flow diagrams of pre-processing and conversion stages
3. Mass and energy balances of pre-processing and conversion stages

1. Calculation of annual forest residues requirement

The parameters used in the calculations of annual forest residues requirement are summarized in Table 1. It should be noted that we assumed that there was no mass loss in the feedstock collection and transportation stages as well as the biofuel products separation process.

Table 1. Parameters for calculating forest residues requirement

Parameters	Notation	Value	Reference
Annual productivity of total biofuels (million liters per year)	P_v	100	
Mass conversion rate			
Forest residues to bio-oil (kg/kg dry forest residues)	α_{frtb}	0.367	[1]
Forest residues to wood pellet (kg/kg dry forest residues)	α_{frtw}	0.89	[2]
Wood pellet to bio-oil (kg/kg dry wood pellet)	α_{wptb}	0.367[a]	[1]
bio-oil to deoxygenated oil (wt%)	α_{botd}	75	[1]
Moisture content (wet basis, wt%)			
Wood pellet	MC_{wp}	5.6	[2]
Biofuels products distribution (wt%)			
Gasoline	β_g	21	[3]
Jet fuel	β_{jf}	25	[3]
Diesel	β_d	35	[3]
Heavy oil	β_{ho}	19	[3]
Biofuels density[b] (kg/m^3)			
Gasoline	ρ_g	739	[4]
Jet fuel	ρ_{jf}	808	[4]
Diesel	ρ_d	843	[4]
Heavy oil	ρ_{ho}	944	[4]

[a] Assume wood pellet has the same conversion rate as forest residues to bio-oil

[b] Assume each type of biofuel has the same density as corresponding conventional petroleum fuel

Forest residues requirement for Fr-CIR, Bo-DBR, Wp-CIR scenarios (R_i, dry tonne/yr, *i*={Fr-CIR, Bo-DBR, Wp-CIR}) can be calcualted by Equation (1) and Equation (2):

When *i*=Fr-CIR, Bo-DBR:

$$R_i = \frac{P_v \times \rho_{bm}}{\alpha_{botd} \times \alpha_{frtb}} \tag{1}$$

When *i*=Wp-CIR:

$$R_i = \frac{P_v \times \rho_{bm}}{(\alpha_{botd} \times \alpha_{wptb} \times \alpha_{frtw})(1 - MC_{wp})} \tag{2}$$

Where ρ_{bm} is the density of biofuels mix in kg/m^3, and ρ_{bm} can be calculated by Equation (3):

$$\rho_{bm} = \frac{\sum_j \beta_j}{\sum_j \frac{\beta_j}{\rho_j}}, \qquad j = \{g, jf, d, ho\} \tag{3}$$

The total feedstock requirement is apportioned among four feedstock delivery points (FDPs) according to their feedstock availability and proximity to Chevron oil refinery, that is, the closer the FDP to the refinery, the higher priority it will be given for forest residues utilization. The annual forest residues supply at each FDP for different scenario is shown in Table 2.

Table 2. Annual forest residues supply at each FDP for different scenarios

	Forest residues supply (dry tonne/yr)		
	Fr-CIR	Bo-DBR	Wp-CIR
Chilliwack	7.64E+04	7.64E+04	7.64E+04
Squamish	3.14E+04	3.14E+04	3.14E+04
Powell River	1.21E+05	1.21E+05	1.21E+05
Port Alberni	7.12E+04	7.12E+04	1.07E+05
Total	3.00E+05	3.00E+05	3.36E+05

2. Stream flow diagrams of pre-processing and conversion stages

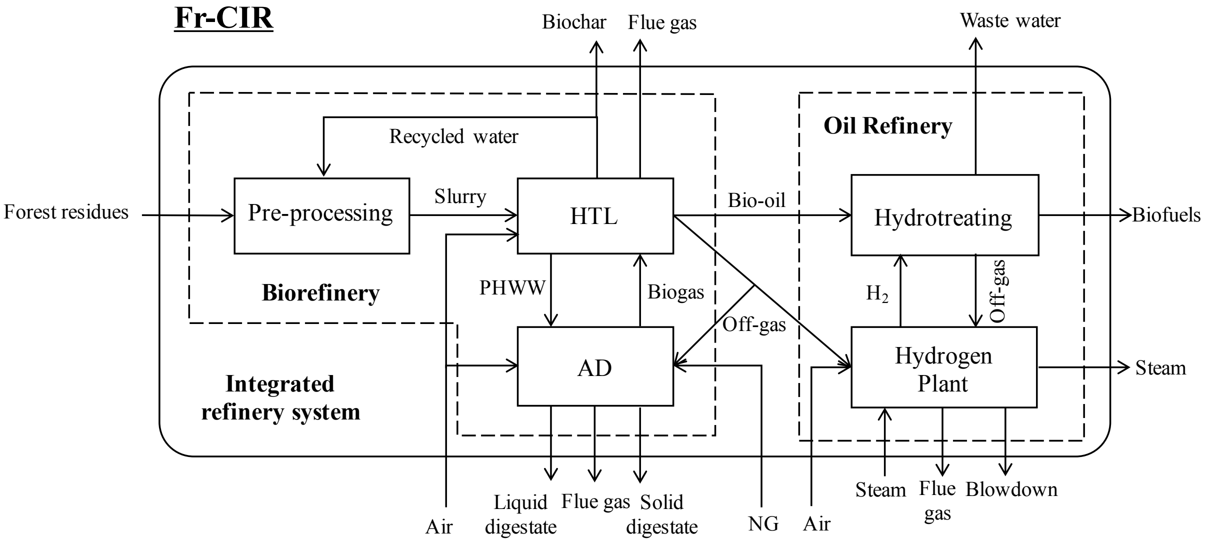

Figure 1. The streams flow diagram of Fr-CIR scenario

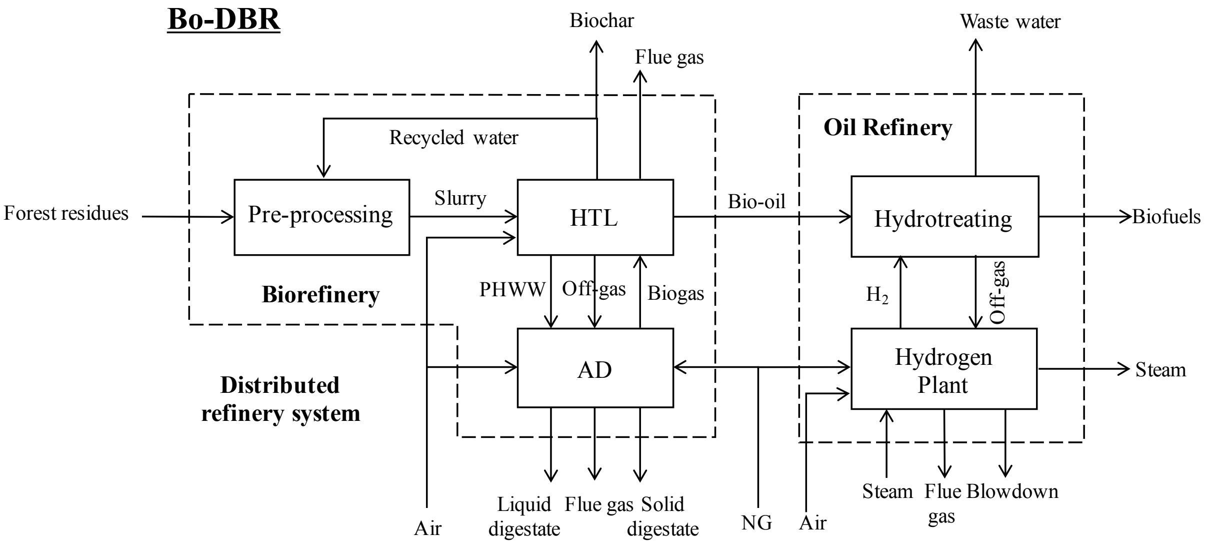

Figure 2. The streams flow diagram of Bo-DBR scenario

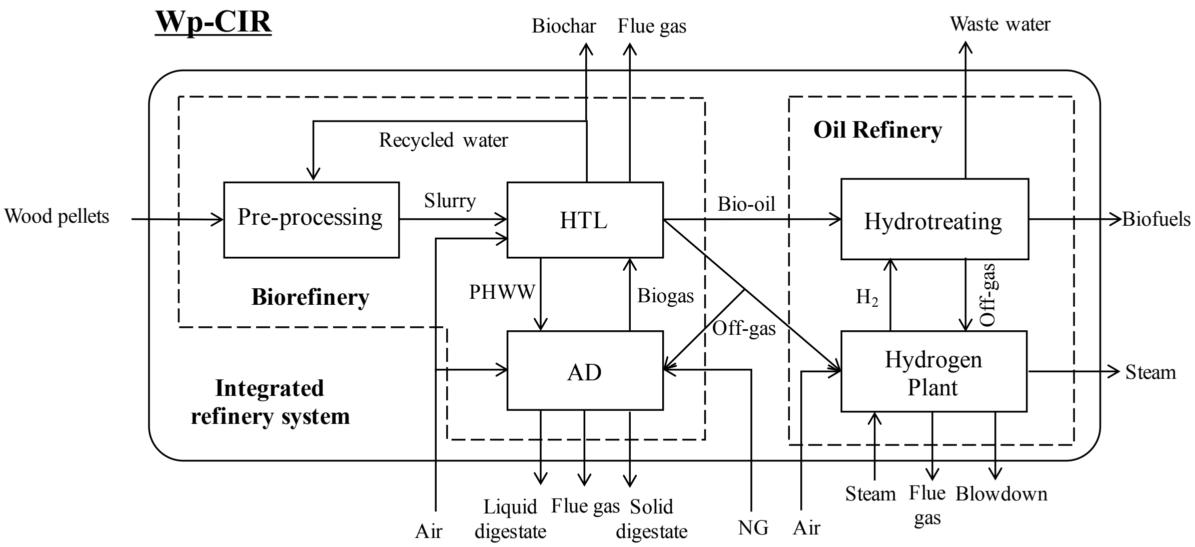

Figure 3. The streams flow diagram of Wp-CIR scenario

3. Mass and energy balances of pre-processing and conversion stages

The mass and energy balances of all operation units in pre-processing and conversion stages are tabulated as below:

3.1 Pre-processing stage

Table 1. Mass balance of pre-processing for each scenario

	Mass balance (kg/s)		
Input	Fr-CIR	Bo-DBR	Wp-CIR
Forest residues[a] / Wood pellet[b]	20.41	20.41	11.01
Recycled water	109.93	109.93	118.88
**Total**	**130.34**	**130.34**	**129.89**
Output			
Biomass slurry	130.34	130.34	129.89
**Total**	**130.34**	**130.34**	**129.89**

[a] for Fr-CIR and Bo-DBR scenarios;
[b] for Wp-CIR scenario

Table 2. Energy balance of pre-processing for each scenario

	Energy balance (MW)		
Input	Fr-CIR	Bo-DBR	Wp-CIR

Forest residues[a] / Wood pellet[b]	191.98	191.98	192.08
Power	2.86	2.86	2.85
Recycled water	36.88	36.88	39.88
**Total**	**231.72**	**231.72**	**234.81**
Output			
Biomass slurry	231.72	231.72	234.81
**Total**	**231.72**	**231.72**	**234.81**

[a] for Fr-CIR and Bo-DBR scenarios

[b] for Wp-CIR scenario

3.2 Conversion stage

3.2.1 HTL unit

Table 3. Mass balance of HTL for each scenario

	Mass balance (kg/s)		
Input	Fr-CIR	Bo-DBR	Wp-CIR
Biomass slurry	130.34	130.34	129.89
Biogas from AD	3.33	3.33	3.32
Combustion air	17.07	17.07	17.02
**Total**	**150.75**	**150.75**	**150.23**
Recycled			
Off-gases	0.2	0.2	0.2
Output			
Bio-oil	3.83	3.83	3.83
Recycled water	109.93	109.93	118.88
PHHW	14.20	14.20	4.80
Biochar	0.58	0.58	0.58
Off-gas	1.80	1.60	1.59
Flue gas	20.41	20.61	20.54
**Total**	**150.75**	**150.75**	**150.23**

Table 4. Energy balance of HTL for each scenario

	Energy balance (MW)		
Input	Fr-CIR	Bo-DBR	Wp-CIR
Biomass slurry	231.72	231.72	234.81
Power	4.03	4.03	4.10
Biogas from AD	49.20	49.20	49.03
**Total**	**284.96**	**284.96**	**287.94**

Recycled			
Off-gases	1.21	1.21	1.21
Output			
Bio-oil	148.52	148.52	148.52
Recycled water	36.88	36.88	39.88
PHWW	61.15	61.15	62.84
Biochar	11.68	11.68	11.64
Off-gas	9.50	9.50	9.47
Heat loss	17.23	17.23	15.58
**Total**	**284.96**	**284.96**	**287.94**

3.2.2 Anaerobic digestion (AD) unit

Table 5. Mass balance of AD for each scenario

	Mass balance (kg/s)		
Input	Fr-CIR	Bo-DBR	Wp-CIR
PHWW	14.20	14.20	4.80
NG	0.08	0.00	0.08
Combustion air	1.37	0.00	1.38
**Total**	**15.65**	**14.20**	**6.26**
Output			
Biogas	3.33	3.33	3.32
Solid digestate	0.18	0.17	0.18
Liquid digestate	10.78	10.79	1.30
Flue gas	1.45	0.00	1.46
**Total**	**15.75**	**14.20**	**6.26**

Table 6. Energy balance of AD for each scenario

	Energy balance (MW)		
Input	Fr-CIR	Bo-DBR	Wp-CIR
Off-gas from HTL	3.48	9.50	3.45
PHWW	61.15	61.15	62.84
Power	5.03	5.03	5.02
NG	4.22	0.00	4.32
**Total**	**70.40**	**75.69**	**67.86**
Output			
Biogas	49.20	49.20	49.03
Heat loss	12.03	17.31	17.72
Liquid digestate	9.17	9.17	1.11
**Total**	**70.40**	**75.69**	**67.86**

3.2.3 Hydrotreating unit

Table 7. Mass balance of hydrotreating for each scenario

	Mass balance (kg/s)		
Input	Fr-CIR	Bo-DBR	Wp-CIR
Bio-oil	3.83	3.83	3.83
Hydrogen	0.13	0.13	0.13
**Total**	**3.96**	**3.96**	**3.96**
Output			
Biofuels	2.62	2.62	2.62
Waste water	1.06	1.06	1.06
Off-gas	0.28	0.28	0.28
**Total**	**3.96**	**3.96**	**3.96**

Table 8. Energy balance of hydrotreating for each scenario

	Energy balance (MW)		
Input	Fr-CIR	Bo-DBR	Wp-CIR
Bio-oil	148.52	148.52	148.52
Hydrogen	18.49	18.49	18.49
Power	1.12	1.12	1.12
**Total**	**167.01**	**167.01**	**167.01**
Output			
Biofuels	120.22	120.22	120.22
Waste water	30.69	30.69	30.88
Off-gas	16.11	16.11	16.11
**Total**	**167.01**	**167.01**	**167.01**

3.2.4 Hydrogen production unit

Table 9. Mass balance of hydrogen plant for each scenario

	Mass balance (kg/s)		
Input	Fr-CIR	Bo-DBR	Wp-CIR
NG (feed)	0.00	0.12	0.00
Off-gas (feed)	0.92	0.24	0.92
Off-gas (fuel)	0.37	0.04	0.37
Combustion air	3.01	3.03	3.01
Steam required	1.26	1.26	1.26
**Total**	**5.56**	**4.69**	**5.56**
Recycled			

off-gases	0.91	0.91	0.91
Output			
Steam produced	1.81	1.81	1.81
Flue gas	3.15	2.27	3.15
Hydrogen	0.13	0.13	0.13
Blowdown	0.48	0.48	0.48
**Total**	**5.56**	**4.69**	**5.56**

Table 10. Energy balance of hydrogen plant for each scenario

	Energy balance (MW)		
Input	Fr-CIR	Bo-DBR	Wp-CIR
NG (feed)	0.00	6.02	0.00
Off-gas (feed)	19.94	13.92	19.94
Off-gas (fuel)	2.19	2.19	2.19
Steam required	3.53	3.53	3.53
Power	0.15	0.15	0.15
**Total**	**25.81**	**25.81**	**25.81**
Recycled			
off-gases	5.81	5.81	5.81
Output			
Hydrogen	18.49	18.49	18.49
Heat loss	2.26	2.26	2.26
Steam produced	5.06	5.06	5.06
**Total**	**25.81**	**25.81**	**25.81**

Reference

1. Tews IJ, Zhu Y, Drennan CV, Elliott D., Snowden-Swan LJ, Onarheim K, et al. Biomass direct liquefaction options: technoeconomic and life cycle assessment. Richland; 2014.
2. Pa A. Development of British Columbia wood pellet life cycle inventory and its utilization in the evaluation of domestic pellet applications. University of British Columbia; 2010.
3. Elliott D, Hallen R, Schmidt A. DOE Bioenergy Technologies Office (BETO) 2015 project peer review hydrothermal processing of biomass. PNNL. 2015. http://energy.gov/sites/prod/files/2015/04/f22/thermochemical_conversion_hallen_222301.pdf. Accessed 4 Apr 2016.
4. Delucchi M, Levelton. GHGenius v4.03. 2013. http://www.ghgenius.com/. Accessed 7 Sep 2015.
