# Supplementary material for: Life-cycle assessment of transportation biofuels from hydrothermal liquefaction of forest residues in British Columbia
Source: Biotechnol Biofuels. 2018 Feb 3;11:23. doi: 10.1186/s13068-018-1019-x (PMC5797420; doi:10.1186/s13068-018-1019-x)
Supplement: Supplementary file 3 — Additional file 3. Equipment energy input and process emission factors. [file 13068_2018_1019_MOESM3_ESM.pdf]

Additional file 3 - Equipment energy input and processes emission factors

This file summarizes the equipment energy input factors and all processes emissions factors used in building the life cycle inventory of HTL biofuels. The equipment energy input factors cover the equipment used in biomass collection and transportation and pre-processing stages, while the emissions factors cover all life cycle stages, including biomass collection and transportation, pre-processing, conversion, biofuels distribution and end use. Besides, a section is added to describe the emission factors associated with by-product biochar application. Emission factors are classified into two types: upstream and downstream. The upstream emission factors account for emissions associated with the upstream supply chain of materials and energy, i.e., production and delivery, while the downstream emission factors are related to the emissions from the materials and energy utilization.

1. Biomass collection and transportation

The emissions from biomass collection and transportation stages are mainly contributed by the operation of diesel-powered and marine diesel-powered equipment. The upstream is associated with diesel production and delivery, and the downstream is related to diesel combustion in the equipment engine. The energy input factors for the diesel-powered equipment are presented in

Table 1. The upstream and downstream emission factors are presented in Table 2 to Table 4.

Table 1. Equipment energy input for biomass collection and transportation modeling

Equipment	Fuel type	Process	Energy input	Reference
Loader	Diesel	Load forest residues to chipper	0.82 L/dry tonne	[1]
Chipper	Diesel	Chip forest residues	3.01 L/dry tonne	[1]
Dump truck	Diesel	Shuttle chipped forest residues to FDP	6279 KJ/Tkm	[2]
Loader	Diesel	Load feedstock at FDPs to STs or LTTs	1.02 L/dry tonne	[1]
ST	Diesel	Transport forest residues or wood pellets to refinery	1988 KJ/Tkm	[2]
LTT	Diesel	Transport bio-oil to refinery	1988 KJ/Tkm	[2]
Ferry	Marine diesel	Carry loaded STs or LTT on the sea	95 L/km	[3]

Table 2. Diesel and marine diesel production and delivery emission factors

Pollutants	Diesel[a] kg/MJ diesel	Marine diesel[b] kg/MJ marine diesel
CO_2	2.05E-02	2.05E-02
NMOCs	4.10E-06	4.10E-06
CH_4	1.31E-04	1.31E-04
CO	1.36E-05	1.36E-05
N_2O	6.00E-07	6.00E-07
NO_2	5.17E-05	5.17E-05
SO_x	4.46E-05	4.46E-05
PM	3.06E-06	3.06E-06

[a] from [2], "Upstream Results HHV | Hwy diesel, crude oil" model

[b] from [2], "Upstream Results HHV | Marine/Rail diesel, crude oil" model

Table 3. Biomass collection equipment operation emission factors

Pollutants	Chipper and loader on forest stands[a] kg/dry tonne biomass	Dump truck[b] kg/Tkm	Loader at feedstock delivery points[c] kg/MJ diesel
CO_2	1.17E+01	4.35E-01	6.82E-02
NMOCs	7.10E-03	4.50E-05	2.15E-05
CH_4	1.15E-02	2.70E-05	1.10E-06
CO	1.02E-01	7.50E-05	9.60E-05
N_2O	0.00E+00	1.90E-05	2.86E-05
NO_2	2.13E-01	1.64E-04	1.41E-04
SO_x	1.18E-02	1.50E-05	6.54E-07
PM	1.12E-03	8.00E-06	0.00E+00

[a] from [1]

[b] from [2], "Freight Emissions | Medium Duty Truck" model

[c] from [2], "Equip Emis Factors | Wheeled loader, diesel powered" model

Table 4. Transportation emission factors

Pollutants	Semi-trailer[a] kg/Tkm	Liquid tanker truck[a] kg/Tkm	Ferry[b] kg/tonne diesel
CO_2	1.38E-01	1.38E-01	3.49E+03
NMOCs	1.43E-05	1.43E-05	2.64E+00
CH_4	8.45E-06	8.45E-06	1.87E-01
CO	2.38E-05	2.38E-05	8.14E+00
N_2O	5.94E-06	5.94E-06	1.28E+00
NO_2	5.19E-05	5.19E-05	6.67E+01
SO_x	4.62E-06	4.62E-06	2.20E+01
PM	2.54E-06	2.54E-06	1.32E+00

[a] from [2], "Freight Emissions | Heavy Duty Truck" model

[b] from [3], the total emission factors of ferry are the sum of underway, maneuvering and dockside emission factors

2. Pre-processing

The emissions from biomass pre-processing stage are contributed by the operation of equipment in biorefinery, including diesel-powered front-end loader, electricity-powered grinder and auxiliary equipment, as well as the operation of wood pellet plant in Wp-CIR scenario. The upstream is associated with diesel and electricity production and delivery, and the downstream is related to diesel combustion in equipment engines. It should be noted that the emission factors of wood pellet plant operation are presented in a life cycle basis, which cover both the upstream and downstream emissions. Besides, the emission factors of electricity generation in Alberta is summarized blow, which is used in the sensitivity analysis.

The energy input factors for the equipment used in feedstock pre-processing stage are presented in Table 5. The upstream emission factors of diesel are the same as those presented in

Biomass collection and transportation section (see Table 2) and the downstream emission factors of diesel combustion in front-end loader in biorefinery are same as the emission factors of loader operation at feedstock delivery points (see Table 3). The emission factors of wood pellet plant operation and electricity generation are presented in Table 6 to Table 10.

Table 5. Energy input of feedstock pre-processing in biorefinery

Equipment	Fuel type	Process	Energy input	Reference
Front-end loader	Diesel	Unload biomass feedstock	0.42 L/dry tonne biomass	[4]
Grinder	Electricity	Grind biomass feedstock	71.2 kWh/dry tonne biomass	[4]
Auxiliary equipment	Electricity	Chip cleaning, dust collection	5 kWh/dry tonne biomass	[4]

Table 6. Wood pellet plant operation Emission factors[a]

Pollutants	kg/tonne of wood pellet
CO_2	8.33E+00
NMOC	1.32E-02
CH_4	6.05E-02
CO	2.95E-01
N_2O	6.40E-03
NO_2	1.58E-01
SO_x	1.89E-02
PM	2.07E-01

[a] from Pa et al. (2012)

Table 7. British Columbia (BC) electricity mix profile and electricity generation and distribution efficiency

	Natural gas (Boiler)	Natural gas (Turbine)	Fuel oil	Biomass	Hydro	Wind
Contribution to BC electricity[a]	1.43%	1.43%	1.52%	4.91%	90.44%	0.28%
Fuel to electricity efficiency[b]	42.34%	42.34%	44.66%	20.00%	100.00%	100.00%
Electricity distribution efficiency[c]	92.00%	92.00%	92.00%	92.00%	92.00%	92.00%

[a] from [6], the average value of 2010-2012, and assume electricity generated by natural gas (boiler) and natural gas (turbine) share 50% of total natural gas generation, respectively.

[b] from [6]

[c] from [2] , "Elec Emissions"

Table 8. British Columbia (BC) electricity generation emission factors[a]

	Natural gas(Boiler)			Natural gas(Turbine)			Fuel oil			Biomass			Hydro			Wind			BC electricity Mix
	Up-stream	Down-stream	Total	Up-stream	Down-stream	Total	Up-stream	Down-stream	Total	Up-stream	Down-stream	Total	Up-stream	Down-stream	Total	Up-stream	Down-stream	Total	
Pollutants	kg/MJ electricity distributed																		
CO2	1.59E-2	1.33E-1	1.49E-1	1.59E-2	1.21E-1	1.37E-1	3.42E-2	1.91E-1	2.26E-1	-	-	-	3.26E-3	4.53E-3	7.79E-3	-	-	3.32E-3	1.46E-2
NMOCs	6.40E-6	3.90E-6	1.03E-5	6.40E-6	6.30E-7	7.03E-6	8.35E-6	2.94E-6	1.13E-5	-	1.93E-5	1.93E-5	-	-	-	-	-	-	1.37E-6
CH4	1.71E-4	2.57E-6	1.73E-4	1.71E-4	8.93E-6	1.80E-4	2.95E-4	2.17E-6	2.97E-4	-	3.47E-5	3.47E-5	-	2.72E-4	2.72E-4	-	-	-	2.57E-4
CO	1.34E-5	9.40E-5	1.07E-4	1.34E-5	8.51E-5	9.85E-5	2.01E-5	3.87E-5	5.88E-5	-	9.92E-4	9.92E-4	-	-	-	-	-	-	5.25E-5
N2O	3.66E-7	9.32E-8	4.59E-7	3.66E-7	3.11E-6	3.48E-6	6.48E-7	3.55E-8	6.83E-7	-	1.54E-5	1.54E-5	-	-	-	-	-	-	8.21E-7
NOx	9.80E-5	1.72E-4	2.70E-4	9.80E-5	1.82E-4	2.80E-4	9.97E-5	1.91E-4	2.91E-4	-	3.64E-4	3.64E-4	-	-	-	-	-	-	3.01E-5
SOx	1.26E-5	1.40E-6	1.40E-5	1.26E-5	1.28E-6	1.38E-5	6.76E-5	2.07E-4	2.75E-4	-	3.46E-4	3.46E-4	-	-	-	-	-	-	2.15E-5
PM	6.63E-7	8.51E-6	9.17E-6	6.63E-7	6.85E-6	7.52E-6	3.38E-6	3.48E-5	3.81E-5	-	1.32E-4	1.32E-4	-	-	-	-	-	-	7.31E-6

[a] from [2] , "Elec Emissions"

Table 9. Alberta electricity mix profile and electricity generation and distribution efficiency

	Natural gas (Boiler)	Natural gas (Turbine)	Fuel oil	Coal	Hydro	Wind
Contribution to Alberta electricity[a]	9.78%	9.78%	0.93%	72.40%	3.53%	3.58%
Fuel to electricity efficiency[b]	33.17%	33.17%	26.10%	29.65%	100.00%	100.00%
Electricity distribution efficiency[c]	92.00%	92.00%	92.00%	92.00%	92.00%	92.00%

[a] from [6], the average value of 2010-2012, and assume electricity generated by natural gas (boiler) and natural gas (turbine) share 50% of total natural gas generation, respectively.

[b] from [6] , “Elec Emissions”

[c] from [2] , “Elec Emissions”

Table 10. Alberta electricity generation emission factors[a]

	Natural gas(Boiler)			Natural gas(Turbine)			Fuel oil			Coal			Hydro			Wind			AB electricity Mix
	Up-stream	Down-stream	Total	Up-stream	Down-stream	Total	Up-stream	Down-stream	Total	Up-stream	Down-stream	Total	Up-stream	Down-stream	Total	Up-stream	Down-stream	Total	
Pollutants	kg/MJ electricity distributed																		
CO2	2.03E-2	1.33E-1	1.53E-1	2.03E-2	1.21E-1	1.41E-1	5.86E-2	1.91E-1	2.50E-1	1.55E-2	2.82E-1	2.97E-1	3.26E-3	4.53E-3	7.79E-3	-	-	3.32E-3	2.47E-1
NMOCs	8.17E-6	3.90E-6	1.21E-5	8.17E-6	6.30E-7	8.80E-6	1.43E-5	2.94E-6	1.72E-5	1.09E-6	2.69E-6	3.78E-6	-	-	-	-	-	-	4.94E-6
CH4	2.18E-4	2.57E-6	2.21E-4	2.18E-4	8.93E-6	2.27E-4	5.05E-4	2.17E-6	5.07E-4	1.02E-4	2.76E-6	1.05E-4	-	2.72E-4	2.72E-4	-	-	-	1.34E-4
CO	1.71E-5	9.40E-5	1.11E-4	1.71E-5	8.51E-5	1.02E-4	3.44E-5	3.87E-5	7.31E-5	8.01E-6	3.45E-5	4.25E-5	-	-	-	-	-	-	5.23E-5
N2O	4.67E-7	9.32E-8	5.60E-7	4.67E-7	3.11E-6	3.58E-6	1.11E-6	3.55E-8	1.14E-6	5.56E-7	1.18E-7	6.74E-7	-	-	-	-	-	-	9.04E-7
NOx	1.25E-4	1.72E-4	2.97E-4	1.25E-4	1.82E-4	3.07E-4	1.71E-4	1.91E-4	3.62E-4	4.82E-6	8.21E-4	8.26E-4	-	-	-	-	-	-	6.60E-4
SOx	1.60E-5	1.40E-6	1.74E-5	1.60E-5	1.28E-6	1.73E-5	1.16E-4	2.07E-4	3.23E-4	2.41E-6	4.21E-4	4.23E-4	-	-	-	-	-	-	3.13E-4
PM	8.47E-7	8.51E-6	9.35E-6	8.47E-7	6.85E-6	7.70E-6	5.78E-6	3.48E-5	4.05E-5	1.30E-6	5.83E-5	5.96E-5	-	-	-	-	-	-	4.52E-5

[a] from [2] , “Elec Emissions

3. Conversion

The emissions from conversion stage are contributed by the following processes:

- Upstream processes:

The production and delivery of materials, including HTL buffer agent Na_2CO_3, natural gas (NG) as heating fuel for anaerobic digestion (AD), hydrotreating catalyst NiMo/Al_2O_3, hydrogen production catalyst NiMo/Al_2O_3. Additionally, for Bo-DBR scenario, NG is required as feedstock for hydrogen production. Upstream emission factors are summarized in Table 11.

The production and delivery of energy, i.e., electricity. See Table 8 for BC electricity mix emission factors.

- Downstream processes:

The combustion of NG for heating AD, the combustion of biogas for heating HTL, hydrogen production via steam reforming using NG as feedstock (Bo-DBR scenario), hydrogen production via steam reforming using off-gases from HTL and hydrotreating as feedstock (Fr-CIR and Wp-CIR scenarios). Downstream emission factors are summarized in Table 12.

Table 11. Materials production and delivery emission factors of conversion stage

Pollutants	Na_2CO_3[a] (HTL) kg/kg Na_2CO_3	NG[d] (AD) kg/MJ NG	NiMo/Al_2O_3 (Hydrotreating)[b] kg/kg NiMo/Al_2O_3	NiMo/Al_2O_3 (Hydrogen production)[c] kg/kg NiMo/Al_2O_3
CO_2	6.94E-01	6.55E-03	9.37E-01	3.35E+00
NMOCs	9.63E-05	3.27E-06	6.53E-05	3.90E-04
CH_4	7.59E-04	1.49E-04	7.85E-04	7.81E-03
CO	5.40E-04	6.57E-06	3.28E-04	1.42E-03
N_2O	1.17E-06	2.15E-07	5.82E-06	5.29E-05
NO_2	4.23E-04	3.92E-05	3.65E-04	2.37E-03
SO_x	4.50E-04	5.37E-06	1.69E-04	3.42E-03
PM	1.72E-04	4.36E-07	6.72E-05	3.30E-04

[a] from [7], "Soda ash production for use in US" model, with US electricity mix changed to BC electricity mix
[b] from [7], "Mo/Ni spent catalyst-biobased" model, with US electricity mix changed to BC electricity mix
[c] from [7], "Mo/Ni spent catalyst-petrochemical" model, with US electricity mix changed to BC electricity mix

[a] from [2], “Upstream Results HHV | CNG, NG” model, also applied for NG as feedstock in Bo-DBR scenario

Table 12. Downstream emission factors of conversion stage

Pollutants	AD heating[a] (NG as fuel) kg/MJ NG	HTL heating[b] (biogas as fuel) kg/MJ biogas	Hydrogen production[c] (NG as feedstock) kg/MJ NG	Hydrogen production[d] (off-gases as feedstock) kg/MJ off-gases
CO_2	5.02E-02	0.00E+00	5.02E-02	0.00E+00
NMOCs	3.69E-06	3.69E-06	9.72E-06	9.72E-06
CH_4	9.74E-07	9.74E-07	3.79E-07	3.79E-07
CO	3.56E-05	3.56E-05	7.58E-06	7.58E-06
N_2O	4.91E-07	4.91E-07	2.37E-07	2.37E-07
NO_2	6.19E-05	6.19E-05	1.90E-05	1.90E-05
SO_x	2.67E-07	2.67E-07	9.48E-08	9.48E-08
PM	3.22E-06	3.22E-06	2.84E-06	2.84E-06

[a] from [2], “Equip Emis Factors | Industrial boiler | NG” model

[b] from [2], “Equip Emis Factors | Industrial boiler | NG” model. CO_2 emission is modified to be 0 since the carbon in biogas is biogenic.

[c] from [2], “Equip Emis Factors | Hydrogen Production Plants | NG” model

[d] from [2], “Equip Emis Factors | Hydrogen Production Plants | NG” model. CO_2 emission is modified to be 0 since the carbon in off-gases is biogenic.

4. Biofuels distribution and end use

The emissions from biofuel distribution stage are contributed by the operation of diesel-powered liquid tanker truck (distribution of gasoline, diesel and heavy oil) and electricity-powered pipeline (distribution of jet fuel). The upstream is associated with diesel and electricity production and delivery, and the downstream is related to diesel combustion in the truck engine. The upstream and downstream emission factors of diesel are the same as those presented in the biomass collection and transportation stage (see Table 2 and Table 4, respectively). The pipeline transportation consumes electricity from BC grid and the energy consumption for transporting 1 tonne of biofuel via pipeline for 1 km is 404 Btu [8], which is equivalent to 0.292 MJ/Tkm. The BC electricity mix emission factors are presented in Table 8.

The emissions from biofuels end use stage are associated with biofuels combustion in the

vehicle and airplane engines, which are summarized in Table 13 as follow:

Table 13. Biofuels combustion in vehicle and jet engines emission factors

Pollutants	Gasoline[a] kg/MJ	Jet[b] kg/MJ	Diesel[c] kg/MJ	Heavy oil[d] kg/MJ
CO_2	2.87E-04	0.00E+00	7.31E-04	0.00E+00
NMOCs	4.07E-05	1.08E-06	5.78E-06	2.94E-05
CH_4	3.28E-06	1.68E-06	3.79E-06	7.15E-07
CO	1.64E-03	7.00E-06	7.71E-06	1.91E-04
N_2O	1.32E-06	1.90E-06	2.96E-06	1.99E-06
NO_2	3.56E-05	2.50E-04	2.45E-05	1.79E-03
SO_x	1.20E-06	0.00E+00	2.07E-06	6.76E-07
PM	2.32E-06	2.50E-06	9.60E-07	3.58E-05

[a] from [2], "LDV Summ | Biomass Fuels, Gasoline, Wood Res" model. The emission factors are presented in unit of g/km originally, and they are converted to kg/MJ base by assuming that the energy intensity of light duty vehicle (LDV) is 2.21 MJ/(person*km) and average 3 persons are transported.

[b] from [2], "Freight Emissions | Airplanes, BTL, Wood Res" model. The emission factors are presented in unit of g/Tkm originally, and they are converted to kg/MJ base by assuming that the energy intensity of airplane is 15 MJ/Tkm.

[c] from [2], "HDV Summ | Biomass Fuels, FT Diesel, Wood Res" model. The emission factors are presented in unit of g/km originally, and they are converted to kg/MJ base by assuming that the fuel efficiency of heavy duty vehicle (HDV) is 40 L/100km and higher heating value of diesel is 38.65 MJ/L.

[d] from [2], "Freight Emissions | Marine Liquids and Bulk Freight, Fuel oil (0.002% S), Crude oil" model. The emission factors are presented in unit of g/Tkm originally, and they are converted to kg/MJ base by assuming that the energy intensity of marine vessel is 60 KJ/Tkm. Besides, the CO_2 emission in the original model is modified to be 0 as the carbon in biofuels are biogenic.

5. Biochar application

Biochar produced from HTL of forest residues as a by-product was assumed to be shipped out to a hypothetic farm for soil amendment. The emissions associated with the biochar application are transportation emissions, which use the same upstream and downstream emission factors as those described in Biomass collection and transportation stage (see Table 2 and Table 4). Meanwhile, the application of biochar can create greenhouse gas emissions reduction credit. According to the assumption made by Roberts et al. [9], 80% of the carbon in biochar can be viewed as stable sequestered carbon. Besides, N in biochar was assumed to displace the same

amount of nitrogen fertilizer, so the upstream emissions of nitrogen fertilizer can be avoided. Table 14 tabulates the emission factors associated with the production and delivery of nitrogen fertilizer.

Table 14. Nitrogen fertilizer production and delivery emission factors[a]

Pollutants	kg/kg N fertilizer
CO_2	4.47E+00
NMOCs	1.55E-03
CH_4	7.80E-03
CO	7.39E-03
N_2O	2.01E-02
NO_2	1.94E-02
SO_x	1.89E-02
PM	3.90E-03

[a] from [10], "Nitrogen fertilizer, as N {GLO}|market for |Alloc Def, S" model

Reference

1. Johnson L, Lippke B, Oneil E. Modeling biomass collection and woods processing life-cycle analysis. For Prod J. 2012;62:258.
2. Delucchi M, Levelton. GHGenius v4.03. 2013. http://www.ghgenius.com/. Accessed 7 Sep 2015.
3. British Columbia Ministry of Environment. 2014/2015 B.C. best practices methodology for quantifying greenhouse gas emissions. Victoria; 2014.
4. Tews IJ, Zhu Y, Drennan CV, Elliott D., Snowden-Swan LJ, Onarheim K, et al. Biomass direct liquefaction options: technoeconomic and life cycle assessment. Richland; 2014.
5. Pa A, Craven JS, Bi XT, Melin S, Sokhansanj S. Environmental footprints of British Columbia wood pellets from a simplified life cycle analysis. Int J Life Cycle Assess. 2012;17:220–31.
6. Environment Canada. National Inventory Report 1990-2013. Greenhouse gas sources and sinks in Canada. 2015.
7. Argonne National Laboratory. The Greenhouse Gases, Regulated Emissions, and Energy Use in Transportation (GREET) Model. 2015. https://greet.es.anl.gov/. Accessed 9 Mar 2016.
8. Dunn JB, Elgowainy A, Vyas A, Lu P, Han J, Wang M, et al. Update to transportation parameters in GREET. 2013.
9. Roberts KG, Gloy BA, Joseph S, Scott NR, Lehmann J. Life cycle assessment of biochar systems: Estimating the energetic, economic, and climate change potential. Environ Sci Technol. 2010;44:827–33.
10. Wernet G, Bauer C, Steubing B, Reinhard J, Moreno-Ruiz E, Weidema B. The ecoinvent database version 3 (part I): overview and methodology. Int J Life Cycle Assess. 2016;21:1218–30.
